# Kisspeptin levels in infertile and miscarried women: a cross-sectional study on the relationship with methylenetetrahydrofolate reductase gene polymorphisms and biochemical parameters

**DOI:** 10.1590/1806-9282.20250716

**Published:** 2025-10-17

**Authors:** Adem Keskin, Naim Uzun, Recai Aci, Melek Bilgin, Taner Karakaya, Ozlem Sezer, Samet Semiz

**Affiliations:** 1 Aydın Adnan Menderes University, Faculty of Medicine, Department of Medical Biochemistry – Aydın, Turkey.; 2 Ağrı İbrahim Çeçen University, Faculty of Pharmacy, Department of Clinical Pharmacy – Ağrı, Turkey.; 3 Aydın Adnan Menderes University, Soke Vocational School of Health Services, Department of Medical Services and Techniques – Aydın, Turkey.; 4 Samsun University, Faculty of Medicine, Department of Microbiology – Samsun, Turkey.; 5 Samsun University, Faculty of Medicine, Department of Medical Genetics – Samsun, Turkey.; 6 Ondokuz Mayıs University, Graduate Education Institute, Department of Medical Biology – Samsun, Turkey.

**Keywords:** Infertility, Miscarriage, Kisspeptin, Gene polymorphism, Homocysteine

## Abstract

**OBJECTIVE:**

Low kisspeptin levels are observed in those with reproductive difficulties. This study was designed to examine and compare kisspeptin levels in women with infertility and miscarriages. Moreover, the aim of this study was to evaluate the possible relationship between kisspeptin levels and the C677T and A1298C polymorphisms of the methylenetetrahydrofolate reductase gene, as well as certain biochemical parameters in these two groups.

**METHODS:**

A total of 76 women, including 46 infertile women and 30 women with a history of miscarriages, were included in the study. In addition to kisspeptin levels, thyroid-stimulating hormone, free triiodothyronine, free thyroxine, ferritin, vitamin B12, folate, prolactin, and homocysteine levels were analyzed in individuals in both groups. The distribution of the C677T and A1298C polymorphisms of the methylenetetrahydrofolate reductase gene was also evaluated.

**RESULTS:**

Women with infertility had lower kisspeptin levels (p=0.019, Cohen's d=0.62) and higher vitamin B12 levels (p=0.018, Cohen's d=0.50) than women with miscarriages. Thyroid-stimulating hormone, free triiodothyronine, free thyroxine, ferritin, folate, prolactin, and homocysteine levels were not different between the two groups. There was no difference in the genotype distribution of methylenetetrahydrofolate reductase C677T and A1298C gene polymorphisms between the groups. There was no correlation between kisspeptin levels and other parameters in both groups. No difference was found between kisspeptin levels of individuals with different methylenetetrahydrofolate reductase C677T and A1298C gene polymorphism genotypes.

**CONCLUSION:**

Kisspeptin levels were lower in women with infertility compared to women with a history of miscarriages, suggesting a possible association between low kisspeptin levels and reproductive difficulties and warranting further investigation.

## INTRODUCTION

Infertility, defined as the inability to conceive despite 12 months of regular unprotected sexual intercourse, affects approximately 12.7% of women of reproductive age in the United States, and those affected receive treatment every year^1^. Infertility in women, which accounts for about 70% of infertility cases, continues to increase every year, and the underlying factors need to be further investigated^2^. However, dysfunction in the hypothalamic–pituitary–ovarian axis, a tightly regulated system that controls the female reproductive system, leads to ovulation disorders^3^.

Miscarriage is typically described as the loss of a pregnancy before the fetus reaches viability. Globally, approximately 23 million miscarriages occur each year, which equates to about 44 pregnancy losses per minute. On average, the risk of miscarriages is estimated to be 15.3% among recognized pregnancies. Additionally, the prevalence of women who have experienced at least one miscarriage is around 10.8%^4^. Miscarriage, also known as spontaneous termination of pregnancy before 12 weeks (early miscarriage) or between 12 and 24 weeks (late miscarriage), can have significant physiological and psychological effects on the patient. It is also associated with significant healthcare costs^5^. However, some factors that may influence the feedback regulation of the hypothalamic–pituitary–adrenal axis may be associated with pregnancy outcomes in women with recurrent miscarriages^6^.

Kisspeptin is a neuropeptide that plays a fundamental role in the control of reproduction and fertility. It has profound effects on the reproductive system by regulating hormone release and contributes to healthy fertility^7^. Specialized nerve cells in the hypothalamus region of the brain that secrete the neuropeptide kisspeptin stimulate gonadotropin-releasing hormone (GnRH) neurons to regulate the coordinated release of gonadotropins. This process is vital for the continuation of the estrous cycle and the occurrence of ovulation. It also plays an important role in the regulation of placentation during pregnancy^8^. Kisspeptin levels, which are abundantly expressed in syncytiotrophoblasts, increase significantly during healthy pregnancy. On the other hand, changes in kisspeptin levels are associated with an increased risk of adverse maternal and fetal complications^9^.

Changes in kisspeptin levels, which play a critical role in the reproductive system and have regulatory effects during pregnancy, may be associated with adverse pregnancy outcomes. Accordingly, this study aimed to evaluate the potential effects of this neuropeptide on reproductive health by comparing kisspeptin levels in two different groups with reproductive difficulties (infertile and miscarrying women). It also aims to investigate the possible association of differences in kisspeptin levels with some biochemical parameters and methylenetetrahydrofolate reductase (MTHFR) gene polymorphism analyzed in infertile and miscarried women.

## METHODS

### Study design

The necessary ethics committee decision for the study was received from Samsun University Clinical Research Ethics Committee (protocol number GOKAEK 2024/8/2, approval date: 03.06.2024). The number of participants included in the study was determined using the G*Power 3.1.9.7 power analysis program {test family: t-tests; statistical test: Difference between two independent means [two groups]; type of power analysis: a priori: required sample size with given α, power, and effect size [Cohen's d=0.8, α=0.05, and power (1-β)=0.95]}. After ethics committee approval, 46 infertile women and 30 women with a history of miscarriages who applied to Samsun University Faculty of Medicine Genetics Outpatient Clinic between July 1, 2024, and December 24, 2024, were included in the study. Within the scope of the inclusion criteria, women who could not achieve pregnancy despite unprotected and regular sexual intercourse for at least 1 year for the infertile group and women with a history of at least one or more miscarriages for the miscarriage group were included in the study. In addition, being between 18 and 45 years of age, agreeing to participate in the study, and signing the informed consent form were determined as the basic requirements for both groups. Exclusion criteria included individuals with genetic or chromosomal diseases, hormonal or metabolic diseases (e.g., diabetes and thyroid disorders), and anatomical disorders of the reproductive organs; patients who have undergone or are undergoing cancer treatment; and individuals with missing medical data required for study participation. After the purpose of the study was explained and an informed consent form was obtained, blood samples were collected from the participants.

### Analysis of methylenetetrahydrofolate reductase C677T (Ala222Val, rs1801133) and A1298C (Glu429Ala, rs1801131) polymorphisms

DNA was isolated from participants’ blood samples using a Sigma-Aldrich (Taufkirchen, Germany) DNA extraction kit. MTHFR C677T and A1298C gene polymorphisms were examined using a polymerase chain reaction (PCR)-based restriction fragment length polymorphism assay. In cases where the results were equivocal, a second PCR run was performed for confirmation purposes.

### Biochemical analyses

Blood samples taken from the participants were also analyzed for thyroid-stimulating hormone (TSH), free triiodothyronine (FT3), free thyroxine (FT4), ferritin, vitamin B12, folate, prolactin, homocysteine, and kisspeptin. TSH, FT3, FT4, ferritin, vitamin B12, and folate levels were analyzed on the Roche Cobas 6000 automated analyzer (Roche Diagnostics, Mannheim, Germany). Homocysteine levels were measured using the chemiluminescence immunoassay technique on a Cobas 601 automated analyzer (Roche Diagnostics, Mannheim, Germany). Participants’ kisspeptin levels were analyzed using the Human kisspeptin 1 ELISA kit (Catalog no. YLA0302HU) manufactured by Shanghai YL Biotech (Zhangjiang Town, Pudong New Area, Shanghai) on an Architect I2000SR immunoanalyzer (Abbott Laboratories, IL, USA).

### Statistical analysis

SPSS for Windows 22.0 program was used for statistical analyses. Continuous variables were presented as mean±standard deviation (X±SD) and categorical variables as percentage and frequency. The value of p<0.05 was considered statistically significant. The normality of distribution of continuous variables obtained from the study was assessed with the Shapiro-Wilk test, and the homogeneity of variances was assessed with the Levene test. Based on the results of these tests, the independent samples t-test was used to compare continuous variables, and the chi-square test was used to compare categorical variables. In addition, Pearson's correlation analysis was used to evaluate the relationship between variables found to be significant between the two groups.

## RESULTS

The mean age of the participants in the infertile group and the miscarriage group was 31.22±5.56 years and 28.33±4.69 years, respectively. Laboratory findings and MTHFR C677T and MTHFR A1298C genotype distributions of the two groups are presented in Table 1.

**Table 1:** Laboratory findings and methylenetetrahydrofolate reductase C677T and methylenetetrahydrofolate reductase A1298C genotype distributions of infertile and miscarriage groups.

Parameter		Infertile, n=46	Miscarriage, n=30	p
TSH (mIU/L)		2.09±1.69	1.73±0.93	0.300
FT3 (pg/mL)		3.14±0.49	3.14±0.41	0.978
FT4 (ng/dL)		1.20±0.17	1.26±0.2	0.188
Ferritin (μg/L)		37.53±41.88	27.91±20.85	0.247
Vitamin B12 (pg/mL)		442.84±231.63	347.67±103.40	0.018
Folate (ng/mL)		8.35±5.55	7.74±5.51	0.638
Prolactin (ng/mL)		20.21±12.02	20.49±12.18	0.931
Homocysteine (μmol/L)		9.74±3.17	10.56±2.58	0.254
Kisspeptin (ng/L)		145.61±87.06	210.98±130.38	0.019
MTHFR C677T	CC, n (%)	24 (52.17)	13 (43.33)	0.309
	CT, n (%)	19 (41.3)	12 (40)	
	TT, n (%)	3 (6.52)	5 (16.67)	
MTHFR A1298C	AA, n (%)	21 (45.65)	15 (50)	0.685
	AC, n (%)	15 (32.61)	10 (33.33)	
	CC, n (%)	10 (21.74)	5 (16.67)	

TSH: thyroid-stimulating hormone; FT3: free triiodothyronine; FT4: free thyroxine; MTHFR: methylenetetrahydrofolate reductase. CC, CT, and TT refer to genotypes of the C677T polymorphism. CC, AA and AC refer to genotypes of the A1298C polymorphism.

Kisspeptin levels in the infertile group were found to be lower than those in the miscarriage group (p=0.019, Cohen's d=0.62) (Table 1). Vitamin B12 levels in the infertile group were also found to be higher than those in the miscarriage group (p=0.018, Cohen's d=0.50) (Table 1). No significant difference was found between the two groups in other laboratory findings (Table 1). Additionally, no significant difference was found in the MTHFR C677T and MTHFR A1298C genotype distributions of the two groups (Table 1).

Correlation analysis was performed to determine the relationship between kisspeptin levels and vitamin B12 levels in both groups, but no significant correlation was found. Additionally, the kisspeptin levels of participants with different MTHFR C677T and MTHFR A1298C genotypes were compared, but no significant difference was found.

## DISCUSSION

The discovery of kisspeptin as a critical central regulatory factor of GnRH release has given humans a new understanding of neuroendocrine regulation in the human reproductive system. Kisspeptin regulates female follicle development, oocyte maturation, and ovulation via the hypothalamic–pituitary–gonadal axis by promoting GnRH secretion. Defects in kisspeptin and its receptor system can lead to clinical symptoms such as female infertility, central precocious puberty, and idiopathic hypogonadotropic hypogonadism^10^. Fertility is supported by increased kisspeptin levels. Additionally, women experiencing infertility have lower kisspeptin levels compared to fertile women^11^. However, kisspeptin has been used as a trigger in in vitro fertilization treatment and has been associated with positive results^12^.

Ultrasound scanning and/or β-human chorionic gonadotropin (β-hCG) measurement is the most commonly used method to diagnose miscarriage, the most common complication during pregnancy, especially in the first trimester. However, plasma kisspeptin is a promising biomarker for miscarriages in addition to β-hCG and has better predictive value later in the first trimester^13^. A recent systematic review reported that a highly accurate serum marker needs to be developed to predict viable pregnancy, and kisspeptin is a potential biomarker for this purpose. In addition, kisspeptin may be used as a potential biomarker of pregnancy viability in the near future^14^. A recent meta-analysis of 12 studies involving 2,050 participants reported that low kisspeptin levels were significantly associated with an increased risk of miscarriages and that kisspeptin may be an effective biomarker for early diagnosis^15^. On the other hand, regardless of body mass index, higher serum kisspeptin concentrations have been reported in women with polycystic ovary syndrome who have a high frequency of miscarriages^16^.

In this study, the mean kisspeptin levels of infertile women were found to be lower than those of women who had miscarriages.

Thyroid autoimmunity and thyroid disorders are common among women of reproductive age and have been linked to negative effects on fertility and pregnancy, both in natural conception and assisted reproductive technology treatments. Therefore, it seems reasonable to perform thyroid panel screening in infertile women who aim to have children^17^. Similarly, it is recommended that women with recurrent miscarriages have their thyroid function tests evaluated^18^. In this study, no significant difference was found between the thyroid function test averages of infertile women and women who had miscarriages.

According to the latest green guidelines on recurrent miscarriage from the Royal College of Obstetricians and Gynecologists, routine testing for the MTHFR mutation is not recommended because inherited thrombophilia has a weak association with recurrent miscarriages^18^. On the other hand, a recent study reported that infertility was closely associated with MTHFR gene polymorphisms and homocysteine levels^19^. Similarly, another recent study reported that high homocysteine levels may be considered as a risk factor affecting ovarian reserve in infertile women^20^. Additionally, recurrent miscarriages of unknown cause are associated with homocysteine, vitamin B12, and folate levels^21^. In this study, no significant difference was found between the homocysteine and folate averages of infertile women and women who had miscarriages. In addition, there was no significant difference in the MTHFR C677T and MTHFR A1298C genotype distributions of infertile and miscarriage women. On the other hand, the mean vitamin B12 levels of infertile women were found to be higher than those of women who had miscarriages.

In this study, no significant correlation was found between kisspeptin levels and other biochemical parameters analyzed in both infertile and miscarried women. Furthermore, no significant difference was found between kisspeptin levels of women with different MTHFR C677T and MTHFR A1298C genotypes in both infertile and miscarried women. The sample size may be sufficient to compare kisspeptin levels between the two groups; however, the lack of association with MTHFR polymorphisms may be attributed to insufficient sample size for genetic subgroup analysis. This may be considered as a limitation of this study. The absence of a control group consisting of healthy women giving birth may also be considered as a limitation of this study. On the other hand, the comparison of the kisspeptin results of infertile and aborted women is one of the strengths of this study. The findings suggest a potential role for low kisspeptin levels in women with reproductive problems; however, studies with large samples including healthy women giving birth are needed to determine causality. The current study provides baseline data to guide future studies in this direction.

## CONCLUSION

The lower levels of kisspeptin in women with infertility problems compared to women with a history of miscarriages support a possible role for this neuropeptide in reproductive health. This finding requires larger sample-based studies to elucidate the mechanisms of the association between low kisspeptin levels and reproductive difficulties. Yet, there is no sufficient evidence to directly use kisspeptin levels in clinical practice.

## Data Availability

The datasets generated and/or analyzed during the current study are available from the corresponding author upon reasonable request.
